# Correction: Potential airborne transmission of SARS-COV-2 through bathroom ventilation ducts associated with an outbreak in a residential building in Santander, Spain, 2020

**DOI:** 10.1371/journal.pone.0354250

**Published:** 2026-07-20

**Authors:** The PLOS OneStaff

There are errors in the Author Contributions. The correct contributions are:

**Conceptualization:** Shelly L. Miller, Alberto Garcia, Liangzhu Leon Wang, Zhiqiang Zhai, Jose Ramon Aranda, Ignacio Lombillo, Javier Balbás, Delfín Silió, L. David Higuera.

**Data curation:** Shujie Yan, Jose Ramon Aranda, Fernando González-Candelas, Ernesto Cabrillo, L. David Higuera.

**Formal analysis:** Shujie Yan, Jose Ramon Aranda, Ignacio Lombillo, Javier Balbás, Delfín Silió, L. David Higuera.

**Investigation:** Shelly L. Miller, Shujie Yan, Alberto Garcia, Zhiqiang Zhai, Jose Ramon Aranda, Fernando González-Candelas, Ignacio Lombillo, Javier Balbás, Ernesto Cabrillo, Delfín Silió, L. David Higuera.

**Methodology:** Shelly L. Miller, Shujie Yan, Alberto Garcia, Liangzhu Leon Wang, Zhiqiang Zhai, Jose Ramon Aranda, Ignacio Lombillo, L. David Higuera.

**Project administration:** Shelly L. Miller.

**Resources:** Shelly L. Miller, Shujie Yan, Jose Ramon Aranda.

**Software:** Shujie Yan, Jose Ramon Aranda.

**Supervision:** Shelly L. Miller, Liangzhu Leon Wang.

**Validation:** Shelly L. Miller, Shujie Yan, Jose Ramon Aranda.

**Visualization:** Shelly L. Miller, Shujie Yan, Jose Ramon Aranda, L. David Higuera.

**Writing – original draft:** Shelly L. Miller, Shujie Yan, Alberto Garcia, Jose Ramon Aranda, L. David Higuera.

**Writing – review & editing:** Shelly L. Miller, Shujie Yan, Alberto Garcia, Liangzhu Leon Wang, Zhiqiang Zhai, Jose Ramon Aranda, Ignacio Lombillo, L. David Higuera.

The publisher apologizes for the error.
