# Supplementary material for: Potential airborne transmission of SARS-COV-2 through bathroom ventilation ducts associated with an outbreak in a residential building in Santander, Spain, 2020
Source: PLoS One. 2026 May 12;21(5):e0345041. doi: 10.1371/journal.pone.0345041 (PMC13166949; doi:10.1371/journal.pone.0345041)
Supplement: S1 File — (DOCX) [file pone.0345041.s001.docx]

# S1 File.

# Background and Preparatory Steps for Investigating the COVID-19 Outbreak Related to Bathroom Ventilation Exhaust Ducts

## Initial Actions and Approvals

Before starting the investigation into the potential connection between the COVID-19 outbreak and the building’s bathroom ventilation exhaust duct, several initial steps were taken. Professor Aranda officially submitted a letter to the building's management, specifically the homeowners' association, requesting permission to perform the research. This request received support from most residents, who voted in favor and showed their interest in the investigation. This official document is included in the Supplementary Information. Additionally, some residents have asked about the study, and the completed research will be shared with management after it is published.

## Data Collection and Collaboration

Professor F. Candelas contacted the Regional Ministry of Health to obtain anonymized data on the progression of the COVID-19 outbreak within the building. The data was provided a few weeks after the initial request.

## Public Information and Media Reports

Information about the COVID-19 situation in Santander was publicly available daily on the Regional Ministry of Health’s website; however, many of these online sources are no longer accessible. As a result, much of the relevant information was gathered from statements made by Ministry of Health officials to the media.

## Identification and Tracking of the Outbreak

The first indication of the outbreak cluster within the building came from resident David Higuera, who noticed the pattern on his floor. Media reports stated that the second affected apartment was directly above the first, which helped trace the spread of the outbreak through news stories.
