# Supplementary material for: Potential airborne transmission of SARS-COV-2 through bathroom ventilation ducts associated with an outbreak in a residential building in Santander, Spain, 2020
Source: PLoS One. 2026 May 12;21(5):e0345041. doi: 10.1371/journal.pone.0345041 (PMC13166949; doi:10.1371/journal.pone.0345041)
Supplement: S2 File — (DOCX) [file pone.0345041.s002.docx]

# S2 File.

# Building Permits Cover Letter

Two documents were provided prior to the start of this study, which are the research and building access permits granted by the homeowners' association, following consultation with all residents. These documents are found in the Supplemental Information S3 File and S4 File. All data collected for this study have been anonymized. There were no human or animal trials. The trials consisted solely of evaluating the performance of the vertical duct.
