# Supplementary material for: Potential airborne transmission of SARS-COV-2 through bathroom ventilation ducts associated with an outbreak in a residential building in Santander, Spain, 2020
Source: PLoS One. 2026 May 12;21(5):e0345041. doi: 10.1371/journal.pone.0345041 (PMC13166949; doi:10.1371/journal.pone.0345041)
Supplement: S3 File — (DOCX) [file pone.0345041.s003.docx]

To: External Peer Review Report – PLOS One Manuscript

From: Shelly Miller and co-authors

Date: 1/28/2026

Re: Response Memo to comments on PONE-D-25-62493 **Title: “**Potential Airborne Transmission of SARS-CoV-2 Through Bathroom Ventilation Ducts Associated with an Outbreak in a Residential Building in Santander, Spain, 2020”

**Recommendation:** Minor Revisions

Thank you very much for the detailed comments on our submitted manuscript. We are grateful that you appreciated our interdisciplinary work and recommended minor revisions. Your suggestions have helped us to significantly improve and clarify our work.

Below we provide the Comments from Reviewer in grey font that require our response and edits to the manuscript. Our response is in italic black font and the changes to the manuscript are copied here in blue font.

________________________________________________________________________

**Comments from Reviewer:**

**3. Specific Comments & Critique**

**A. The "Control Group" and Building-Wide Generalization**

**Critique:** The study focuses heavily on the infected vertical stack (Home A/B stack). While this is logical for forensic analysis, the manuscript lacks physical measurements from the other three courtyards (patios) in the building that did not experience outbreaks.

The author’s state:

*"No positive PCR tests were detected among the residents of the homes surrounding the three other patios of the building."*

However, regarding physical measurements, it is noted:

*"Additional measurements in other homes were not possible due to a lack of access."*

**Recommendation:** The authors must address this limitation more explicitly in the **Discussion** section. A critical reader might ask: "Did the other courtyards not have outbreaks because their ventilation worked better, or simply because there was no virus present?" Based on the architectural symmetry implied in the text (*"The building... comprises seven floors with eight homes per level, grouped around four patios"*), it is scientifically sound to assume the airflow physics are identical. The authors should explicitly state that the lack of infection in other courtyards is likely due to the absence of an **Index Case** in those stacks, rather than superior ventilation performance. This distinction is crucial for generalizing the risk to the entire building type, not just the specific duct investigated.

***Response:*** *Thank you for this insightful comment. Your comment is accurate: no index case existed in the other courtyards. Furthermore, all the four courtyards are identical in architecture and have the same bathroom exhaust configuration as the case studied in this article. We agree that we must explicitly state that the lack of infection was likely because there was no index case in those areas of the building. Thus, we have added the following statement on Page 8, under the Testing section:*

“The lack of infection around the other courtyards is likely due to the absence of an Index Case in those homes. PCR tests were conducted in the whole building (not only to the A and B sections of the building; Fig. 2).”

**B. The Exacerbating Role of Kitchen Hoods**

**Critique:** One of the most significant findings of this study—which has profound public health implications—is the counter-intuitive impact of kitchen exhaust fans. The data suggests that operating these fans can be more detrimental than passive ventilation issues.

The manuscript states:

*"airflow into the upper bathroom reaches approximately 2 m/s, and CO2 levels are higher. These results indicate that operating a kitchen hood may enhance the risk of aerosol transmission... more than opening the patio window."*

Furthermore, the mechanism is clearly identified:

*"When exhaust rates are high and makeup air is insufficient, the resulting depressurization may draw air and potentially contaminants from adjacent areas."*

**Recommendation:** This finding deserves greater prominence in the **Abstract** and **Conclusions**. The authors should formulate a specific recommendation for residents in buildings with shared ducts: utilizing powerful kitchen extraction without ensuring adequate make-up air (e.g., opening a street-facing window) can actively suck pathogens from neighbors' apartments.

***Response:*** *Thank you for noticing the importance of this issue. We agree that the use of the kitchen exhaust system deserves a little more attention and focus on the manuscript. We also believe that the work presented in this article will hopefully expand the knowledge and design of proper building ventilation design to consider disease transmission. To address this comment, we have added the following to the* ***Abstract****:*

“Additionally, operating the kitchen exhaust fan can augment the movement of aerosols between occupied spaces increasing the potential for infection.”

*We have added the following to the* ***Conclusions****:*

“Additionally, in buildings with shared ducts, sufficient make‑up air is needed to prevent kitchen exhaust use from drawing air—potentially containing pathogens—from neighboring apartments. This can be achieved by opening a street-facing window.”

**C. Generalization from Single-Floor Measurements**

**Critique:** The environmental boundary conditions for the models were derived from a single apartment on the 4th floor.

*"Environmental measurements were collected in the 4th-floor bathroom... of Home B."*

**Recommendation:** While the CFD and CONTAM models extrapolate this to other floors, the authors should briefly acknowledge in the **Limitations** section that "stack effect" pressure differentials vary by height. Therefore, the infiltration rates on the 1st or 2nd floor might differ slightly in magnitude from the measured 4th floor, though the *direction* of flow (reverse flow) likely remains consistent under the observed conditions.

***Response:*** *Thank you for this comment. Very good point. We have added the following text to the* ***CFD model*** *section on page 14:*

“Note that because "stack effect" pressure differentials vary by height, the infiltration rates on the 1st or 2nd floor might differ in magnitude from the measured 4th floor, though the *direction* of flow (reverse flow) likely remains consistent under the observed conditions.”

*We did not add any text to the CONTAM model section because we did not use the measurements from the 4^th^ floor apartment to derive the model. We used the measurements to verify that the model output was accuracy. A key strength of our study is that the CONTAM model for the Santander building captures real-world multizone airflow, including the stack effect within the shared vertical shaft, enabling us to evaluate aerosol transmission throughout the building.*

**D. CFD Model Simplification**

**Critique:** The authors mention simplifying the geometry for the simulation.

*"The model focused on airflow and CO2 distribution... with some non-essential features simplified to reduce computation time."*

**Recommendation:** Please verify or briefly confirm in the text that these "non-essential features" do not include roughness elements or duct junctions that might significantly induce turbulence or pressure drops, which could affect the vertical propagation speed of the aerosols.

***Response:*** *Thank you so much for this insightful and critical comment. This is correct, as the conditions for our CFD simplified model include the calculated duct roughness (based on the typical roughness of brick and mortar, the duct material used in this building). The duct is a vertical shaft made of brick and mortar, and no duct junctions are implemented in this type of construction. We have added the following statement to Page 14, under the* ***CFD section:***

“This simplified CFD model includes variables such as duct roughness based on the typical roughness average of brick and mortar which is the material used for the construction of the bathroom exhaust.”

**4. Conclusion**

This paper provides valuable evidence supporting airborne transmission via shared infrastructure. The methodology is sound, and the conclusions are supported by the data. I recommend **Minor Revisions** to address the discussion points regarding the unmeasured courtyards and to strengthen the warnings regarding kitchen exhaust operation.

**Status:** Accept with Minor Revisions.

Please let us know if you have any additional questions or comments. Thank you again for your time and your insights provided in this peer review.

Respectfully,

Shelly Miller
