# Supplementary material for: Potential airborne transmission of SARS-COV-2 through bathroom ventilation ducts associated with an outbreak in a residential building in Santander, Spain, 2020
Source: PLoS One. 2026 May 12;21(5):e0345041. doi: 10.1371/journal.pone.0345041 (PMC13166949; doi:10.1371/journal.pone.0345041)
Supplement: S4 File — (DOCX) [file pone.0345041.s004.docx]

## S4 File.

## Calle Nicolas Salmeron 4 Letter
