# Supplementary material for: Potential airborne transmission of SARS-COV-2 through bathroom ventilation ducts associated with an outbreak in a residential building in Santander, Spain, 2020
Source: PLoS One. 2026 May 12;21(5):e0345041. doi: 10.1371/journal.pone.0345041 (PMC13166949; doi:10.1371/journal.pone.0345041)
Supplement: Attachment — Submitted filename: Peer Review Report PONE-D-25-62493.pdf [file pone.0345041.s005.pdf]

PLOS ONE - Peer Review Report

Manuscript Number: PONE-D-25-62493 **Title:** Potential Airborne Transmission of SARS-CoV-2 Through Bathroom Ventilation Ducts Associated with an Outbreak in a Residential Building in Santander, Spain, 2020 **Recommendation:** Minor Revisions

1. Executive Summary

This manuscript investigates a vertically clustered outbreak of SARS-CoV-2 in a multi-family residential building in Santander, Spain. The authors present a compelling case that a shared vertical bathroom ventilation duct (shunt system) served as the conduit for airborne transmission, infecting 15 residents across four apartments. The study employs a robust multi-disciplinary methodology, combining epidemiological tracing, whole-genome sequencing, field environmental measurements, CFD simulations, and CONTAM multi-zone modeling. The findings highlight the critical role of building ventilation defects in disease transmission, particularly in older housing stock.

2. General Assessment

The manuscript is well-written, timely, and addresses a significant intersection between public health and building engineering. The integration of genetic sequencing with engineering airflow analysis provides a high level of credibility to the conclusions.

Strengths:

- **Genetic Confirmation:** The use of genomic epidemiology effectively rules out community transmission. The authors note:

"The sequences derived from the building were similar, with one or two nucleotide differences among them and at least 11 differences from the closest control sequence." This strongly supports the hypothesis of a single internal source.

- **"Negative" Evidence:** The observation that apartments with modified ventilation systems were spared provides excellent real-world validation of the proposed transmission mechanism.

"Occupants of three homes... in which the bathroom ventilation had been modified (by installing an exhaust fan with a no-return flap) did not get infected."

However, there are specific areas regarding the generalization of results and the discussion of "control" areas within the building that require clarification before publication.

3. Specific Comments & Critique

A. The "Control Group" and Building-Wide Generalization

Critique: The study focuses heavily on the infected vertical stack (Home A/B stack). While this is logical for forensic analysis, the manuscript lacks physical measurements from the other three courtyards (patios) in the building that did not experience outbreaks.

The authors state:

"No positive PCR tests were detected among the residents of the homes surrounding the three other patios of the building."

However, regarding physical measurements, it is noted:

"Additional measurements in other homes were not possible due to a lack of access."

Recommendation: The authors must address this limitation more explicitly in the **Discussion** section. A critical reader might ask: "Did the other courtyards not have outbreaks because their ventilation worked better, or simply because there was no virus present?" Based on the architectural symmetry implied in the text (*"The building... comprises seven floors with eight homes per level, grouped around four patios"*), it is scientifically sound to assume the airflow physics are identical. The authors should explicitly state that the lack of infection in other courtyards is likely due to the absence of an **Index Case** in those stacks, rather than superior ventilation performance. This distinction is crucial for generalizing the risk to the entire building type, not just the specific duct investigated.

B. The Exacerbating Role of Kitchen Hoods

Critique: One of the most significant findings of this study—which has profound public health implications—is the counter-intuitive impact of kitchen exhaust fans. The data suggests that operating these fans can be more detrimental than passive ventilation issues.

The manuscript states:

"airflow into the upper bathroom reaches approximately 2 m/s, and CO2 levels are higher. These results indicate that operating a kitchen hood may enhance the risk of aerosol transmission... more than opening the patio window."

Furthermore, the mechanism is clearly identified:

"When exhaust rates are high and makeup air is insufficient, the resulting depressurization may draw air and potentially contaminants from adjacent areas."

Recommendation: This finding deserves greater prominence in the **Abstract** and **Conclusions**. The authors should formulate a specific recommendation for residents in buildings with shared ducts: utilizing powerful kitchen extraction without ensuring adequate make-up air (e.g., opening a street-facing window) can actively suck pathogens from neighbors' apartments.

C. Generalization from Single-Floor Measurements

Critique: The environmental boundary conditions for the models were derived from a single apartment on the 4th floor.

"Environmental measurements were collected in the 4th-floor bathroom... of Home B."

Recommendation: While the CFD and CONTAM models extrapolate this to other floors, the authors should briefly acknowledge in the **Limitations** section that "stack effect" pressure differentials vary by height. Therefore, the infiltration rates on the 1st or 2nd floor might differ slightly in magnitude from the measured 4th floor, though the *direction* of flow (reverse flow) likely remains consistent under the observed conditions.

D. CFD Model Simplification

Critique: The authors mention simplifying the geometry for the simulation.

"The model focused on airflow and CO2 distribution... with some non-essential features simplified to reduce computation time."

Recommendation: Please verify or briefly confirm in the text that these "non-essential features" do not include roughness elements or duct junctions that might significantly induce turbulence or pressure drops, which could affect the vertical propagation speed of the aerosols.

4. Conclusion

This paper provides valuable evidence supporting airborne transmission via shared infrastructure. The methodology is sound, and the conclusions are supported by the data. I recommend **Minor Revisions** to address the discussion points regarding the unmeasured courtyards and to strengthen the warnings regarding kitchen exhaust operation.

Status: Accept with Minor Revisions.
